# Endophytic *Bacillus atrophaeus* CHGP13 and salicylic acid inhibit blue mold of lemon by regulating defense enzymes

**DOI:** 10.3389/fmicb.2023.1184297

**Published:** 2023-06-13

**Authors:** Saba Maalik, Anam Moosa, Faisal Zulfiqar, Muhammad Naveed Aslam, Tahir Mahmood, Kadambot H. M. Siddique

**Affiliations:** ^1^Department of Plant Pathology, Faculty of Agriculture and Environment, The Islamia University of Bahawalpur, Bahawalpur, Punjab, Pakistan; ^2^Department of Horticultural Sciences, Faculty of Agriculture and Environment, The Islamia University of Bahawalpur, Bahawalpur, Punjab, Pakistan; ^3^The UWA Institute of Agriculture, The University of Western Australia, Perth, WA, Australia

**Keywords:** *Penicillium italicum*, resistance inducer, benzoic acid, *Citrus limon*, fruit, postharvest, enzyme activity

## Abstract

Lemons (*Citrus limon* L.) are one of the most economically important and consumed fruit worldwide. The species is vulnerable to several postharvest decay pathogens, of which *Penicillium italicum* associated with blue mold disease is the most damaging. This study investigates the use of integrated management for blue mold of lemon using lipopeptides (LPs) extracted from endophytic *Bacillus* strains and resistance inducers. Two resistance inducers; salicylic acid (SA) and benzoic acid (BA) were tested at 2, 3, 4, and 5 mM concentrations against the development of blue mold on lemon fruit. The 5 mM SA treatment produced the lowest disease incidence (60%) and lesion diameter (1.4 cm) of blue mold on lemon fruit relative to the control. In an *in vitro* antagonism assay eighteen *Bacillus* strains were evaluated for their direct antifungal effect against *P. italicum*; CHGP13 and CHGP17 had the greatest inhibition zones of 2.30 and 2.14 cm. Lipopeptides (LPs) extracted from CHGP13 and CHGP17 also inhibited the colony growth of *P. italicum*. LPs extracted from CHGP13 and 5 mM SA were tested as single and combined treatments against disease incidence and lesion diameter of blue mold on lemon fruit. SA + CHGP13 + PI had the lowest disease incidence (30%) and lesion diameter (0.4 cm) of *P. italicum* on lemon fruit relative to the other treatments. Furthermore, the lemon fruit treated with SA + CHGP13 + PI had the highest PPO, POD, and PAL activities. The postharvest quality analysis of the lemon fruit including fruit firmness, total soluble solids, weight loss, titratable acidity, and ascorbic acid content revealed that the treatment SA + CHGP13 + PI had little effect on fruit quality compared to the healthy control. These findings indicate that *Bacillus* strains and resistance inducers can be used as components of integrated disease management for the blue mold of lemon.

## Introduction

Citrus is an economically and nutritionally important fruit crop worldwide, especially in tropical and subtropical areas (Liu et al., 2012). Lemon (*Citrus limon* L.) is the third most significant species of the genus Citrus after orange and mandarin. Citrus fruits are susceptible to various biotic and abiotic challenges during the postharvest stage (handling, shipping, storing, and marketing; Tian et al., 2020). Citrus production faces significant economic losses before and after harvest, particularly due to postharvest deterioration. The harvested citrus fruits are stored and handled in packing houses to maintain their postharvest life and quality and to reduce decay due to pathogen infection. Postharvest fungal pathogens account for 30–50% of citrus crop losses (Porat et al., 2000).

Injuries inflicted during postharvest handling are the major cause of infection by *Penicillium digitatum* Saccardo (associated with the green mold of citrus fruit) and *P. italicum* Wehmer (associated with blue mold of citrus), allowing these pathogens to enter the fruit (Moosa et al., 2021, 2022). These pathogens are omnipresent and cause significant losses wherever citrus is cultivated (Palou et al., 2001). During postharvest storage, 4°C is the most conducive temperature for developing *P. italicum* infection (Wyatt et al., 1995), a nesting-type pathogen that produces enzymes to macerate fruit tissues, enabling pathogen colonization. The infection intensity of blue mold of citrus increases with fruit ripeness. A high density of inoculum deposited in wounds and 20–25°C temperature increase the infection intensity of blue mold of citrus (Papoutsis et al., 2019).

Blue mold is controlled primarily by applying synthetic fungicides such as thiabendazole and imazalil (Cunha et al., 2018). While synthetic chemical fungicides can control several diseases effectively, their use is restricted for controlling postharvest deterioration due to their high and acute residual toxicity, long degradation period, environmental pollution, and effects on food quality and human health (Tripathi and Dubey, 2004). Moreover, the widespread use of chemical fungicides proliferates fungicide-resistant strains, compromising the effectiveness of these chemicals (Palou et al., 2008). Thus, the exploration of new and safer alternatives to reduce the use of synthetic fungicides is an emerging field of research.

Biological control of plant diseases is a safer alternative to chemicals with no harmful effects on human health or the environment, offering sustainable, affordable, and eco-friendly management for the control of plant pathogens (He et al., 2021). A biological control agent should be genetically stable, effective at low concentrations, able to survive under adverse environmental conditions, inexpensive to formulate and produce, shelf-stable, resistant to common pesticides, compatible with commercial processing practices, and non-pathogenic to human health and the host (Wisniewski and Wilson, 1992).

Several biocontrol agents have been studied and used successfully to prevent postharvest diseases in fruit, including yeasts and bacteria (Janisiewicz and Korsten, 2002; Tian et al., 2002; Sharma et al., 2009). *Bacillus* species are widespread endophytic bacteria with impressive biocontrol potential against several plant diseases (Mahaffee and Kloepper, 1997; Algam et al., 2005), mainly by inhibiting the growth of plant pathogens and promoting systemic resistance in plants (Fira et al., 2018). The antagonistic mechanisms of action of endophytic *Bacillus* species include competition for nutrition and space, production of antimicrobial compounds, direct parasitism, and induced systemic resistance in plants (Farzand et al., 2019a,b), which serve as the foundation for synthesizing commercial bio-formulations to manage plant diseases (Cirvilleri, 2008; Nunes, 2012).

The production of antimicrobial compounds is considered the most significant mechanism for the biological control of plant pathogens (Finking and Marahiel, 2004; Ongena and Jacques, 2008; Zhao et al., 2013). Antifungal lipopeptides (LPs), produced by *Bacillus* species, are the most effective antimicrobial compounds that directly suppress plant pathogens (Farzand et al., 2019a, 2020). Three important LP families (fengycin, iturin, and surfactin) along with some others (bacilysin, bacillibactin, bacillaene, and bacillomycin) largely contribute to the antifungal activity of *Bacillus* species (Farzand et al., 2019a,b). *Bacillus* species have been researched extensively and reported to control citrus fruit diseases*. Bacillus subtilis* and its metabolites are used to control citrus green and blue molds and are categorized as GRAS (Generally Recognized As Safe) biocontrol agents (Waewthongrak et al., 2015).

The induction of resistance in host plants by natural and synthetic resistance inducers is a widely researched area. Applying resistance inducers can boost the natural defense system of fruit to suppress postharvest pathogen attacks. Non-toxic synthetic resistance inducers such as salicylic acid (SA), benzoic acid (BA), jasmonic acid (JA), and methyl jasmonate (MJ), are also considered a safer alternative to synthetic chemicals (Qin et al., 2003). They are an attractive management strategy employing plants’ natural defense mechanisms to suppress the infection of several threatening plant pathogens (Terry and Joyce, 2004; Walters et al., 2005). SA is an important resistance inducer that increases postharvest disease resistance against multiple diseases of fruit crops, including pear, mango, and citrus (Shaat and Galal, 2004). SA and JA are essential for controlling plant growth and development and improving plant disease resistance (Yao and Tian, 2005). The induction of resistance is accompanied by an oxidative burst and the creation of pathogenesis-related (PR) proteins (Moosa et al., 2018) and other antimicrobial substances, such as phytoalexins (Asghari and Aghdam, 2010). The application of resistance inducers modulates the activity of chitinase, peroxidase, and polyphenol oxidase, which play an important role in plant defense (Sardar et al., 2023). Several studies have reported that exogenous SA application significantly improved the resistance to various postharvest fungal diseases (Asghari and Aghdam, 2010; Iqbal et al., 2012; Zhou et al., 2018; Moosa et al., 2019). However, single applications of resistance inducers have significantly lowered the effectiveness compared to synthetic pesticides and do not offer commercially adequate postharvest decay management (Iqbal et al., 2012; Moosa et al., 2019).

Combining resistance inducers with other therapies can offer comparable disease control to synthetic chemicals. An integrated management strategy is more effective and reliable for the control of several diseases (Sukorini et al., 2013; Moosa et al., 2021, 2022). Combined physical, chemical, and biological controls have been used to manage postharvest diseases of citrus as components of integrated disease control programs (Naqvi, 2004). For example, the combination of yeast and medicinal plant extracts suppressed the infection of *P. digitatum* on tangerine fruit (Sukorini et al., 2013). Guo et al. (2014) reported that the combined application of *Cryptococcus laurentii* and MJ inhibited the development of *P. digitatum* on mandarins. Hao et al. (2011) reported that *B. amyloliquefaciens* combined with tea saponin (50 μg ml^−1^) suppressed blue mold, green mold, and sour rot of citrus. While *Bacillus* species and resistance inducers can significantly suppress blue mold of lemon, they cannot replace the use of pesticides as Singletreatments. Therefore, different management strategies must be combined to enhance the suppression of postharvest diseases during storage.

The biochemical characterization of defense enzymes in diseased plants is critical for understanding the basic mechanisms involved in plant defense (Moosa et al., 2019). The regulation of defense enzymes acts as universal defense against biotic and abiotic stresses (Hasanuzzaman et al., 2020; Zulfiqar and Ashraf, 2021) Necrotrophic pathogens can alter the activity of oxidative enzymes like peroxidase (POD) and polyphenol oxidase (PPO; Bowles, 1990). The pathogenesis-related (PR) class of proteins plays a key role in plant defense against pathogen attack and other physiological stresses like low temperature, salt, drought, and heavy metal toxicity (Ballester et al., 2013). Phenylalanine ammonia-lyase (PAL), POD, and PPO are three important PR proteins that play a role in the biosynthesis and oxidation of phenolic chemicals and disease resistance in plants (Livak and Schmittgen, 2001; Bill et al., 2017). Plant disease resistance is linked to POD activity (Hammerschmidt et al., 1981) which increases due to pathogen infection in host plants (Scott-Craig et al., 1995). POD accelerates the last stages of lignin and hydrogen peroxide synthesis (Ma et al., 2012) and plays a significant role in plant growth and development. Campa (1991) reported that POD is linked to chlorophyll degradation and lipid peroxidation in dormant plant tissues. Chlorophyll degradation is accelerated by the presence of phenol molecules (Kato and Shimizu, 1985).

The study was undertaken with the hypothesis that the combined application of resistance inducers and *Bacillus* LPs can provide more effective control of blue mold of lemon than a single application. This study aimed to (a) determine the antifungal efficacy of *Bacillus* LPs and SA (Single and combined) against blue mold of lemon, and (b) assess the activity of defense enzymes and postharvest quality of treated and untreated lemon fruit.

## Materials and methods

### Bacterial and fungal cultures

*Bacillus* strains were obtained from the bacterial culture collection in the Molecular Plant Pathology Laboratory at The Islamia University, Bahawalpur, Pakistan (Table 1; Fatima et al., 2023), which were stored in a 60% glycerol stock solution at −80°C. The bacterial cultures were grown on fresh Luria Bertani (LB) medium, and incubated at 37°C. The fungus *P. italicum* was isolated on potato dextrose agar medium (PDA) from decaying lemon fruit showing symptoms of blue mold and incubated at 25 ± 2°C. Purified cultures were obtained by using the single spore culturing technique, preserved in 30% glycerol stock solution at −80°C, and later retrieved on the PDA medium for further experimentation.

**Table 1:** Bacillus cultures used in this study.

Sr #	Strain code	*Bacillus* species
1	CHGP1	*Bacillus cereus*
2	CHGP2	*Bacillus subtilis*
3	CHGP3	*Bacillus atrophaeus*
4	CHGP4	*Bacillus velezensis*
5	CHGP5	*Bacillus Subtilis*
6	CHGP6	*Bacillus Subtilis*
7	CHGP7	*Bacillus subtilis*
8	CHGP8	*Bacillus cereus*
9	CHGP9	*Bacillus amyloliquefaciens*
10	CHGP10	*Bacillus amyloliquefaciens*
11	CHGP11	*Bacillus licheniformis*
12	CHGP12	*Bacillus thuringiensis*
13	CHGP13	*Bacillus atrophaeus*
14	CHGP14	*Bacillus pumilus*
15	CHGP15	*Bacillus velezensis*
16	CHGP16	*Bacillus amyloliquefaciens*
17	CHGP17	*Bacillus thuringiensis*
18	CHGP18	*Bacillus velezensis*

### Fruit

Mature, disease-free, and uniform-sized lemon fruit cv. ‘Meyer Lemon’ were collected from the Citrus orchard at The Islamia University of Bahawalpur, Pakistan. The samples were disinfected with 1% NaOCl for 2 min and, rinsed with sterilized distilled water. Then the samples were air-dried on sterilized paper towels to remove excess moisture from the fruit surface.

### Assay of resistance inducers

Resistance inducers SA and BA were used to test their effect on *P. italicum* infection on lemon fruit. Aqueous solutions of SA and BA were prepared at different concentrations (2, 3, 4, and 5 mM) based on their molar masses. Sterilized fruit were dipped in aqueous solutions of SA and BA for 10 min and then dried on paper towels. A sterilized needle was used to injure the fruit, making a 5 mm artificial wound, before inoculation with 10 μL (1 × 10^6^ spores ml^−1^) of *P. italicum.* Then, a 10 μl aqueous solution of SA and BA was deposited into these wounds using a micropipette. The fruit was placed in sterilized square plastic boxes and incubated for 7 days at 25 ± 2°C. The experiment was conducted in a completely randomized design (CRD) with ten replications for each treatment, repeated twice under the same conditions. The lesion diameter of blue mold of lemon was measured, and disease incidence was determined using the following formula (Sukorini et al., 2013):


Disease incidence(%)=(No. of infected fruit/ No. of total fruit)×100

### Antagonism assay

The antifungal activity of 18 *Bacillus* species was evaluated in a dual culture assay against *P. italicum*. A 5 mm culture block from 7-day-old pathogen culture was placed at the center of a Petri plate containing PDA and incubated at 25 ± 1°C for 2 days. Sterilized filter paper disks were placed 3 cm from both sides of the plate. Then, 10 μl bacterial culture, grown overnight in LB broth (OD600 = 2.5), was inoculated on both filter paper disks. The plates were incubated for 7 days at 25 ± 1°C. The experiment was performed three times under the same conditions with five replications per treatment.

### Extraction of LPs from *Bacillus* strains

Based on the antagonism assay, the best strains (CHGP13 and CHGP17) were used to extract LPs. Luria Bertani (LB) broth (300 ml) was prepared, inoculated with CHGP13, and placed on a rotary shaker at 180 rpm at 30°C for 3 days. Then the bacterial cultures were centrifuged at 10,000 rpm at 4°C for 15 min to obtain the cell-free supernatant. The supernatant was collected in a sterilized glass beaker and incubated at 4°C for 12 h. After incubation cell-free supernatant was centrifuged at 10,000 rpm for 15 min at 4°C. The supernatant was discarded, and the pellet was collected and dried. The pellet was dissolved in 5 ml HPLC-grade methanol (pH 7) and passed through a 0.22 μm syringe filter to remove impurities.

### Antifungal assay with LPs

To determine the effect of LPs on lemon fruit, uniform-sized, disease-free fruit were selected and surface sterilized. A sterilized needle was used to injure the fruit, making a 5 mm artificial wound, before depositing 10 μl LPs solution in each wound. Then each wound was inoculated with 10 μl spore suspension (1 × 10^6^ spores ml^−1^) of the pathogen. The fruit was placed in sterilized square plastic boxes and incubated at room temperature for 7 days. The experiment was conducted in a CRD, with each treatment replicated ten times, and repeated twice under the same conditions. Disease incidence and severity were determined as per the resistance inducers assay.

### Combined assay with resistance inducer and LPs to control blue mold of lemon

To determine the combined effect of the best resistance inducer and LPs, 5 mM SA and the LPs extracted from CHGP13 were used. Mature, disease-free lemon fruit were surface sterilized and dipped in a 5 mm aqueous solution of SA for 10 min. Next, a sterilized needle was used to injure the fruit, making a 5 mm artificial wound, before inoculating with 10 μl of 5 mM SA solution followed by 10 μl of LP solution using a micropipette. Then each wound received 10 μl spore suspension of *P. italicum*. The fruit was incubated in sterilized square plastic boxes for 7 days at 25 ± 1°C. The experiment was conducted in a CRD with ten replications per treatment, repeated twice under the same conditions. Disease incidence and severity were calculated as per the resistance inducers assay.

### Enzyme assays

#### PAL assay

PAL activity was determined using the method of Martinez-Tellez and Lafuente (1997). The enzyme was extracted from 0.4 g flavedo acetone powder (Sala et al., 2005) in 15 ml of 0.1 M sodium borate buffer (pH 8.8) containing 0.02 M mercaptoethanol at 4°C. The reaction mixture contained 0.1 ml enzyme extract, 1.9 ml of 0.05 M sodium borate buffer (pH 8.8), and 1 ml of 20 mM l-phenylalanine. The absorbance of cinnamic acid was determined at 290 nm after incubation at 40°C for 2 h. PAL activity was calculated on a dry weight basis in nmol cinnamic acid per h per g flavedo acetone powder (nmol h^−1^ g^−1^ fresh weight).

#### POD assay

POD activity was measured using the method of Martinez-Tellez and Lafuente (1997). The enzyme extract was prepared by crushing 0.2 g dry weight of flavedo acetone powder in 6 ml of 100 mM Tris–HCL buffer (pH 8) containing 5 mM mercaptoethanol and centrifuged at 11,000 rpm at 4°C for 30 min. The reaction solution contained 2.15 ml of 10 mM sodium acetate (pH 5.3), comprising guaiacol, 0.25 ml of 0.1% H_2_O_2_, and 0.1 ml supernatant. The mixture was incubated at 30°C for 2 min before measuring the absorbance at 470 nm on a spectrophotometer. One unit of POD activity was expressed as a 470 nm absorbance rise per minute. POD activity was calculated in units of POD min^−1^ g^−1^ flavedo acetone powder.

#### PPO assay

PPO activity was assessed using the method of Martinez-Tellez and Lafuente (1997), with some modifications. The enzyme extract comprised 0.2 g flavedo acetone powder (w/w) mixed in 6 mL of 0.05 M potassium phosphate buffer (pH 7.2) containing 1 M KCL and 5% (w/w) polyvinyl–polypyrrolidone. The solution was centrifuged at 15,000 rpm at 4°C for 10 min. The reaction mixture contained 25 μl enzyme extract and 1.25 ml of 0.02 mM caffeic acid. The rate of increase in absorbance at 410 nm and 30°C was measured to determine PPO activity. At 410 nm, an increase of 0.01 absorbance unit per minute was used to define one unit of PPO activity. Thus, PPO activity was calculated in units of PPO activity per minute per gram of fresh weight.

### Postharvest quality analysis of lemon fruit

To determine the effect of SA and LPs on lemon fruit quality, fruit firmness (N), total weight loss (%), titratable acidity (%), total soluble solids (%), and ascorbic acid content (mg 100 g^−1^) were measured. Firmness was measured using a fruit texture analyzer. The reading on the analyzer was set at zero before placing the fruit on the fruit texture analyzer and held tightly. The flat-end probe (8 mm diameter) was pushed into the fruit at 5 mm s^−1^ to 3 mm, with the penetration (firmness) expressed in Newton. The flat-end probe was cleaned between measurements.

Total soluble solids (TSS) in juice from healthy, disease-infected, and treated fruit was measured using a digital refractometer (Lacey et al., 2009). The fruit was cut in half and squeezed to collect the juice. A few drops of juice were placed on the prism plate of the refractometer, and the reading was recorded. The prism plate was wiped with a clean tissue between measurements.

Each fruit was weighed before treatment and 7 days post-treatment to determine weight loss. Weight loss was calculated as follows:


Weightloss(%)=(A/B)/A×100

where A is fruit weight before treatment and B is fruit weight after treatment.

Titratable acidity was measured by titrating fruit juice with 0.1 M NaOH (Hernández et al., 2006). The NaOH solution was slowly added to the fruit juice using a 25 ml clean burette. The titratability of the fruit juice was recorded with a pH meter by placing the electrode in the fruit juice and recording the point when the fruit pH reached neutrality and the amount of NaOH in the burette. The pH meter electrode was washed with distilled water between measurements.

The ascorbic acid (Vitamin C) content in lemon fruit was determined by titrating the fruit juice with 2,6-dichloro-indophenol. The results were calculated in milligrams of ascorbic acid per 100 g fresh weight (Jones and Hughes, 1983).

### Statistical analysis

The experiments were conducted in a completely randomized design. The data was subjected to Tukey’s HSD test after ANOVA at *p* = 0.05 by using the statistical package Statistix version (Ver. 8.1).

## Results

### Effect of resistance inducers on lesion diameter and disease incidence of blue mold of lemon

The preventive treatment with resistance inducers decreased the lesion diameter and incidence (Table 2). The 5 mM SA decreased the disease incidence of blue mold to 60% and the lesion diameter to 1.4 cm, compared to the infected control treatment. Treatment with 2 mM SA showed 93.3% disease incidence and a 2.6 cm lesion diameter of blue mold. The treatments were significantly different from each other at *p* = 0.05. The 5 mM BA treatment decreased disease incidence to 66.7% and lesion diameter to 1.9 cm relative to the infected control treatment. Healthy control fruit remained asymptomatic.

**Table 2:** Effect of resistance inducers on disease incidence and lesion diameter of blue mold of Lemon.

Treatment	Concentration	Disease incidence (%)	Lesion diameter (cm)
SA	2	93.3 ab	2.6 b
	3	86.7 abc	2.3 c
	4	73.3 abc	1.7 d
	5	60.0 c	1.4 d
BA	2	100.0 a	3.1 a
	3	93.3 ab	2.6 b
	4	80.0 abc	2.2 c
	5	66.7 bc	1.9 d
HC	2	0.0 d	0.0 f
	3	0.0 d	0.0 f
	4	0.0 d	0.0 f
	5	0.0 d	0.0 f
IC	2	100.0 a	3.2 a
	3	100.0 a	3.2 a
	4	100.0 a	3.2 a
	5	100.0 a	3.3 a

The values in columns are an average of 15 replicates, analyzed by Tukey’s HSD test in a CRD design. Mean values followed by the same letter do not significantly differ at *p* = 0.05. SA, salicylic acid; BA, benzoic acid; HC, healthy control; IC, infected control.

### *In vitro* antagonism assay

**Figure 1:**
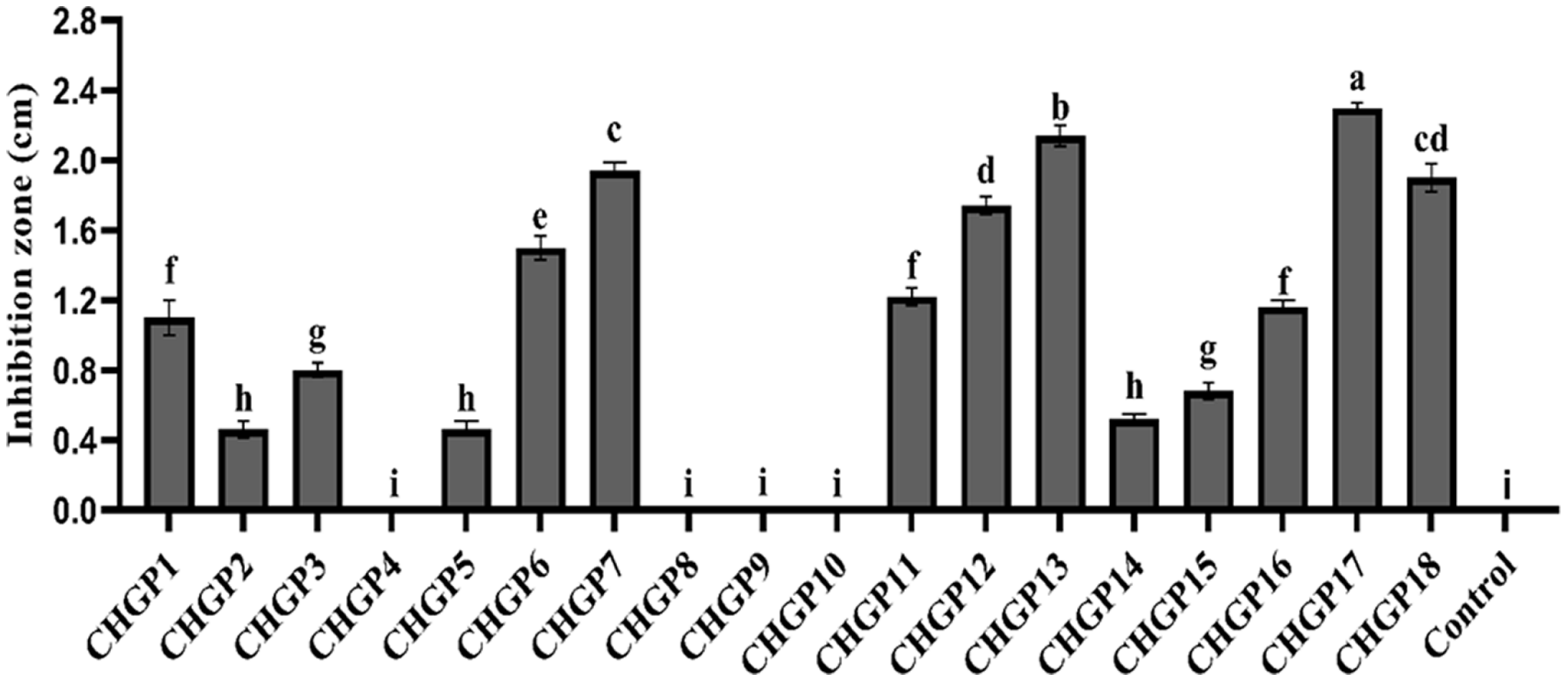
*In vitro* inhibition of *Penicillium italicum* by *Bacillus* strains. Each bar is an average of 15 replicates from three experiments, analyzed by Tukey’s HSD test in a CRD design. Bars with the same letter do not significantly differ at *p* = 0.05. Error bars are the standard error of the mean.

The antifungal effect of 18 *Bacillus* strains against the colony growth of *P. italicum* was tested in a filter paper disk assay. All *Bacillus* strains except CHGP4, CHGP8, CHGP9, and CHGP10 inhibited the colony growth of *P. italicum*. CHGP13 had the greatest inhibition, followed by CHGP17, with inhibition zones of 2.30 and 2.14 cm, respectively (Figure 1). CHGP2, CHGP5, and CHGP14 had the least inhibition, with inhibition zones 0.46, 0.46, and 0.52 cm, respectively.

### Antifungal effect of LPs against *Penicillium italicum*

**Figure 2:**
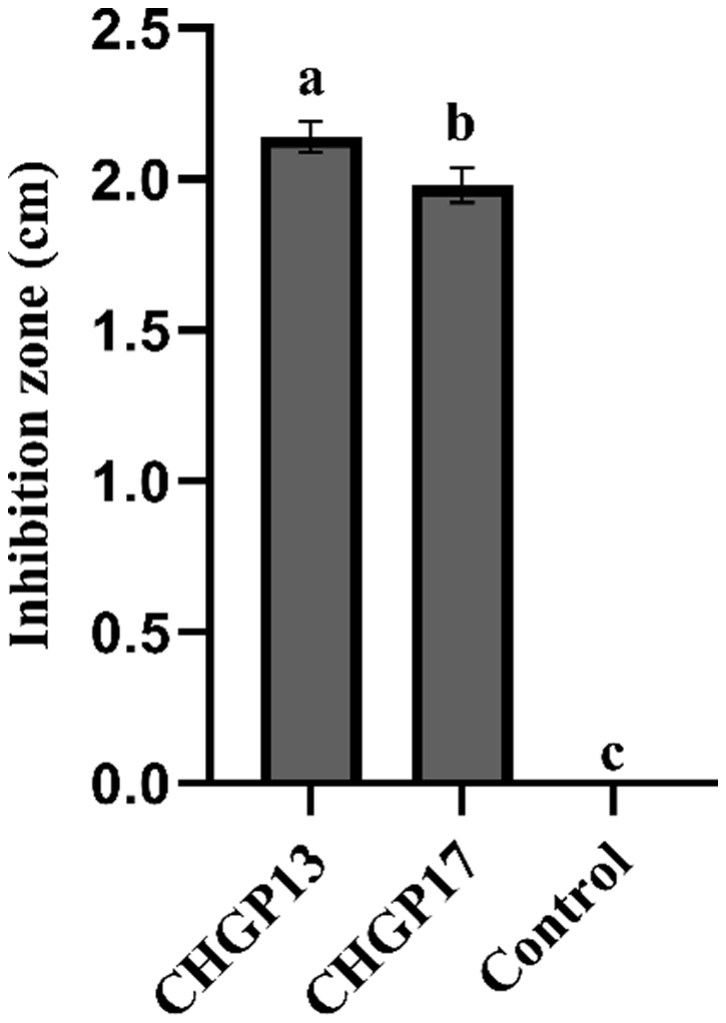
Effect of lipopeptides on *in vitro* colony growth inhibition of *P. italicum.* Each bar is an average of 15 replicates from three experiments, analyzed by Tukey’s HSD test in a CRD design. Bars with different letters significantly differ at *p* = 0.05. Error bars are the standard error of the mean.

LPs were extracted from CHGP13 and CHGP17 and tested against the colony growth of *P. italicum* in an *in vitro* assay. CHGP13 and CHGP17 significantly inhibited *P. italicum* growth compared to the control treatment, with inhibition zones of 2.14 and 1.98 cm, respectively, compared with no inhibition zone in the control treatment (Figure 2).

### Combined assay with resistance inducer and LPs against *Penicillium italicum*

**Figure 3:**
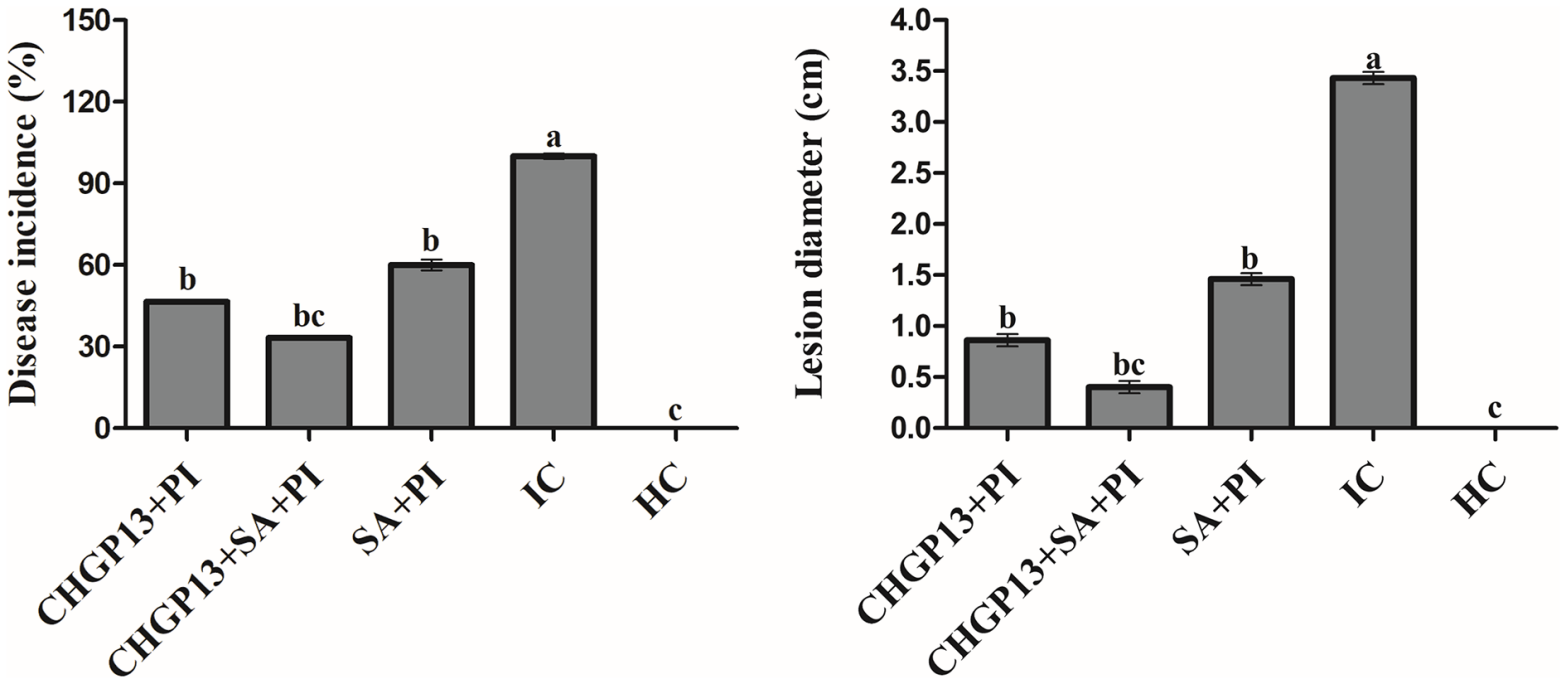
Effect of *Bacillus atrophaeus* CHGP13 and salicylic acid on disease incidence and lesion diameter of blue mold of lemon in a combined assay. Each bar is an average of 30 replicates from three experiments, analyzed by Tukey’s HSD test in a CRD design. Bars with the same letter do not significantly differ at *p* = 0.05. Error bars are the standard error of the mean.

The effect of 5 mM SA and LPs extracted from CHGP13 as single and combined applications was evaluated on the growth inhibition of *P. italicum*. The combined treatment (SA + CHGP13) had the lowest disease incidence and lesion diameter of *P. italicum* compared to the infected control at *p* = 0.05 (Figure 3). Single CHGP13 and SA also decreased disease incidence and lesion diameter but to a lesser degree. The infected control had the highest disease incidence and lesion diameter.

### Assessment of PAL, PPO, and POD activities

**Figure 4:**
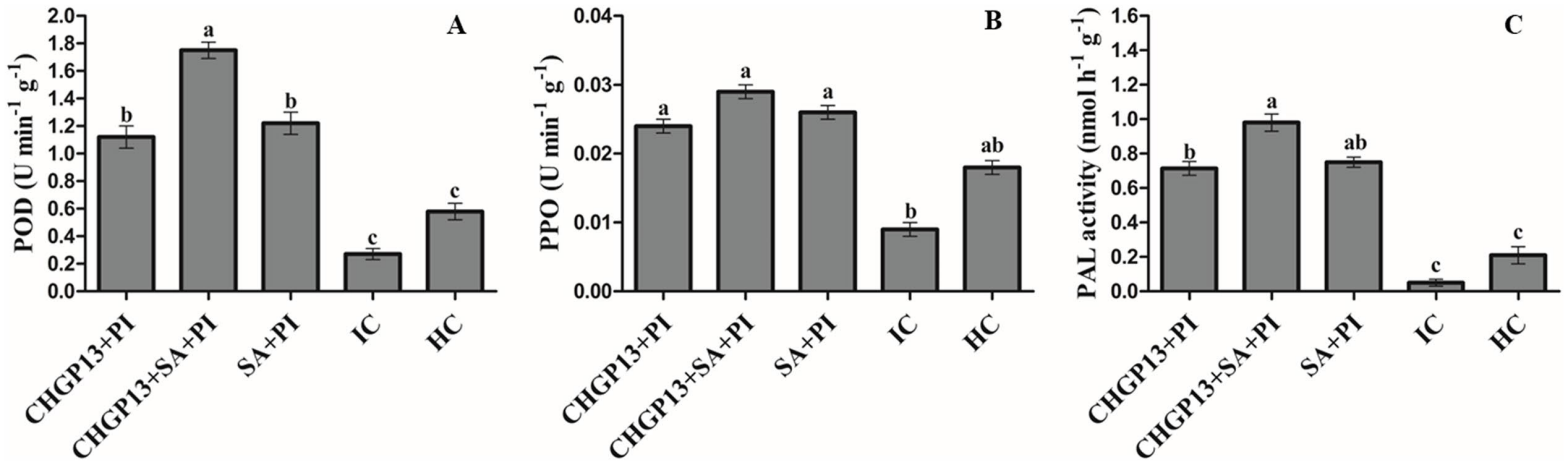
Defense enzyme activities in lemon fruit treated with SA and *B. atrophaeus* CHGP13 as single and combined treatments to suppress blue mold of lemon. **(A)** POD (peroxidase), **(B)** PPO (polyphenol oxidase), and **(C)** PAL (phenylalanine ammonia lyase). Each bar is an average of 15 replicates from three experiments, analyzed by Tukey’s HSD test in a CRD design. Bars with the same letter do not significantly differ at *p* = 0.05. Error bars are the standard error of the mean. CHGP13, *B. atrophaeus* (CHGP13); SA, salicylic acid; PI, *P. italicum*; IC, infected control; HC, healthy control.

The activities of PAL, PPO, and POD in lemon fruit treated with combined and single applications of SA and LPs extracted from CHGP13 were tested. The activity of all tested enzymes increased in fruit treated with Singleor combined application of SA and LPs extracted from CHGP13 relative to control treatments. The combined application of SA and LPs extracted from CHGP13 had the highest PPO, POD, and PAL activities compared to the infected and healthy control treatments at *p* = 0.05 (Figure 4). SA and CHGP13 as single treatments also increased PPO, PAL, and POD activities but to a lesser degree. Fruit infected with *P. italicum* only had the lowest POD, PPO, and PAL activities at *p* = 0.05 analyzed by Tukey’s HSD test (Figure 4).

### Effect on postharvest quality of lemon fruit

**Figure 5:**
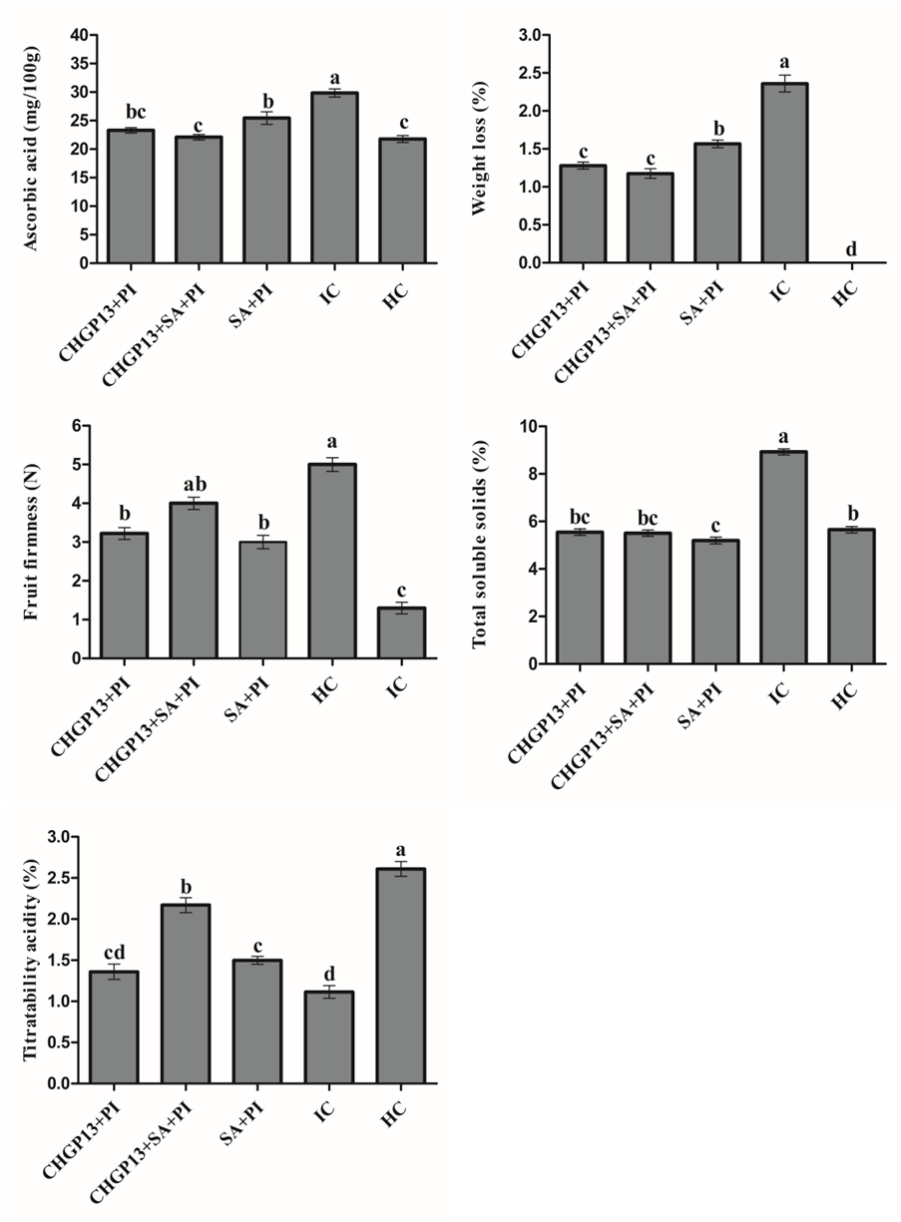
Effect on postharvest quality of lemon fruit after treatment with salicylic acid and lipopeptides extracted from CHGP13 as single and combined treatments. Each bar is an average of 15 replicates from three experiments, analyzed by Tukey’s HSD test in a CRD design. Bars with the same letter do not significantly differ at *p* = 0.05. Error bars are the standard error of the mean. CHGP13, *B. atrophaeus* (CHGP13); SA, salicylic acid; PI, *P. italicum*; IC, infected control; HC, healthy control.

The effect of SA and CHGP13 as single and combined treatments on the postharvest quality of lemon fruit was evaluated. Fruit treated with SA + CHGP13 + PI retained their firmness 7 days post-incubation, while the infected control fruit lost their firmness almost immediately. Fruit treated with SingleSA and CHGP13 also retained their firmness compared to the infected control at *p* = 0.05 analyzed by Tukey’s HSD test. Furthermore, fruit treated with SA + CHGP13 + PI had comparable TSS values to the healthy control treatment 7 days post incubation, with the highest TSS value in infected control fruit at *p* = 0.05. Similarly, lemon fruit treated with SA + CHGP13 had the least weight loss compared to the other treatments. Fruit treated with SA + CHGP13 + PI had comparable titratable acidity and ascorbic acid content to the healthy control treatment at *p* = 0.05 (Figure 5).

## Discussion

Blue mold is a threatening disease causing substantial postharvest losses of citrus fruit (Moosa et al., 2022). Several chemical, physical, and biological control strategies that trigger local and systemic resistance in plants have successfully suppressed blue mold of citrus disease. However, the effectiveness of these treatments is significantly enhanced when combined rather than in single treatments (Moosa et al., 2021, 2022). This study revealed the excellent suppression of blue mold on lemon fruit with the combined application of salicylic acid and lipopeptides extracted from *Bacillus*.

The induction of defense responses in harvested fruit using non-toxic defense elicitors as an alternative to synthetic chemicals is an outstanding and environmentally friendly management approach for managing postharvest pathogens (Moosa et al., 2021). The use of resistance inducers to improve plant defense has garnered a lot of attention in the last decade (Quaglia et al., 2011; Iqbal et al., 2012; Romanazzi et al., 2016; Moosa et al., 2019, 2021). In this study, the effect of resistance inducers at different concentrations was tested on blue mold of lemon fruit, with SA and BA at the highest concentration producing the best results. In another investigation, the inhibitory effect of SA increased with concentration, successfully stimulating the plant defense response (Verberne et al., 2000). Several studies have reported that the suppressive effect of resistance inducers against green and blue mold of lemon increases in a concentration-dependent manner (Iqbal et al., 2012; Romanazzi et al., 2016; Moosa et al., 2019). SA demonstrated suppressive efficacy against many postharvest pathogens of citrus, pear, and mango, according to earlier research reports (Joyce et al., 2001; Shaat and Galal, 2004; Cao et al., 2006; Moosa et al., 2019).

*Bacillus* strains are a good alternative to harmful synthetic chemicals for the biological management of postharvest diseases (Janisiewicz and Korsten, 2002). *Bacillus*-based biopesticide formulations for pre- and post-harvest disease control are predicted to grow in popularity (Fravel, 2005; Droby et al., 2009). The current study intended to investigate the antifungal potential of *Bacillus* strains against lemon blue mould. Biological control using microbial antagonists, particularly *Bacillus* species and yeasts is a promising approach for controlling several fruit diseases (Abraham et al., 2010; Osman et al., 2011; Fatima et al., 2023). *Bacillus* strains tested in this study inhibited the colony growth of *P. italicum* in a direct antagonism assay. Several studies have reported the biological control potential of *Bacillus* species against a variety of citrus fruit diseases (Hao et al., 2011; Tian et al., 2020). In support of this work, Mohammadi et al. (2017) reported that *B. subtilis*, *B. pumilus*, *B. cereus*, and *B. megaterium* suppressed the development of *P. digitatum* on lemon fruit and inhibited mycelial growth and spore germination. In other investigations, the cell-free supernatant of *B. subtilis* showed potential antagonistic activity against *P. digitatum* (Leelasuphakul et al., 2008; Waewthongrak et al., 2015).

Furthermore, the present study was focused on the production of lipopeptides by *Bacillus* species to control the blue mold of lemon. In a filter paper disc assay, LPs extracted from *Bacillus* strains CHGP13 and CHGP17 strongly reduced *P. italicum* growth. *Bacillus* species have antagonistic potential due to the production of a variety of secondary metabolites (Schneider et al., 1999; Stein, 2005), including antibiotics, lipopeptides, low molecular weight volatile compounds, and antifungal proteins (Huang and Chen, 2008; Zhao et al., 2013; Farzand et al., 2019a). The antimicrobial activity of *Bacillus* strains has been linked to the production of LPs in several previous reports (Shoda, 2000; Stein, 2005; Ongena and Jacques, 2008; Farzand et al., 2019a,b; Fatima et al., 2023). The LPs produced by *Bacillus* strains, such as fengycin, iturin, and surfactin, reduced the development of several postharvest diseases, such as gray mold of apple and brown rot of peach (Gueldner et al., 1988; Touré et al., 2004; Ongena et al., 2005). Therefore, the biological control potential of *Bacillus* strains tested in this study can be attributed to the production of antifungal LPs.

*Bacillus* strains and resistance inducers alone do not provide comparable disease control to synthetic chemicals (Walters et al., 2005; Moosa et al., 2019). However, the effectiveness of *Bacillus* strains can be enhanced by combining mixtures of antagonistic agents with other management practices. In the present study, the LPs extracted from *Bacillus* strain CHGP13 combined with SA further reduced the development of blue mold on lemon fruit and the postharvest quality of fruit including ascorbic acid, titratable acidity, fruit firmness, weight loss, and total soluble solids was not affected. In support of this work, Arrebola et al. (2010) revealed that combining *B. amyloliquefaciens* PPCB004 and thyme or lemon grass oil with strong antifungal activity inhibited postharvest pathogens of peach, including *B. cinerea*, *P. expansum*, and *R. stolonifera*, compared to single applications. In another instance, El Hamss et al. (2023) reported that the bioefficacy of *B. amyloliquefaciens* SF14 was enhanced in combination with salicylic acid to suppress green mold of citrus and the treatments were safe to the quality of fruit. Similarly, Zhou et al. (2014) reported that *Pichia membranaefaciens* and salicylic acid were more effective in combination to suppress green mold decay of citrus fruit and did not impair the postharvest quality of fruit. As a result, based on the current findings and multiple earlier investigations, it may be inferred that combining one or more management methods gives superior disease suppression without affecting the postharvest fruit quality.

Furthermore, in order to decipher the defense mechanisms underlying the suppression of blue mold of lemon, the activity of PPO, POD, and PAL enzymes were measured using a spectrophotometer. The activity of PPO, POD, and PAL was highest in lemon fruit treated with combined application of CHGP13 and SA. Resistance induction in harvested fruit is an effective strategy for improving the suppression of fungal pathogens associated with postharvest rots (Cao et al., 2006). According to our findings, SA and CHGP13 boosted the activity of PPO, POD, and PAL in suppressing blue mold infection on lemon. PPO is a critical enzyme in plant defense responses that converts phenols to poisonous quinones, contributing to its high toxicity against a variety of invading pathogens (Mohammadi and Kazemi, 2002; Moosa et al., 2022). Another key defense enzyme is POD, which catalyses the final stage in the synthesis of lignin and transforms phenolic chemicals to highly toxic quinones. Previously, an increase in PPO and POD activity was seen in citrus fruit treated with a combination of *P. membranaefaciens* and salicylic acid (Zhou et al., 2014). The phenolic pathway is regulated by PAL, which catalyses the conversion of phenylalanine to trans-cinnamate. Furthermore, PAL is a precursor to scoparone and scopoletin, both of which have been reported to be antifungal against a variety of fungal diseases (Droby et al., 2002). Our findings revealed a link between increased PPO, POD, and PAL activity and the suppression of blue mold of lemon, implying that these defense enzymes play an important role in plant defence. Several prior studies have found that increase in the activity of PPO, PAL, and POD confers increased resistance to fungal pathogens (Shao et al., 2013; Zhou et al., 2014; Moosa et al., 2021, 2022). Therefore, the current study suggests that PAL, PPO, and POD defence enzymes may be associated with better defense of lemons against blue mold disease.

## Conclusion

In conclusion, the study presents the promising antifungal potential of LPs extracted from *B. atrophaeus* CHGP13 and SA against the development of blue mold of lemon. The combined application of LPs and SA shower higher antifungal activity than the single application by enhancing the activity of defense enzymes PAL, PPO, and POD. Furthermore, the treatments had a very little impact on the postharvest quality of lemon fruit. The underlying mechanisms behind the antifungal potential of SA and LPs must be investigated further before these therapies can be included in the integrated disease management programme for blue mold of lemon.

## Data availability statement

The raw data supporting the conclusions of this article will be made available by the authors, without undue reservation.

## Author contributions

AM conceived the idea of the research. SM and TM performed the experiments. AM, MNA, and FZ supervised the work. SM, AM, and FZ prepared the manuscript draft. AM, FZ, and KHMS reviewed and revised the draft. All authors contributed to the article and approved the submitted version.

## Conflict of interest

The authors declare that the research was conducted in the absence of any commercial or financial relationships that could be construed as a potential conflict of interest.

## Publisher’s note

All claims expressed in this article are solely those of the authors and do not necessarily represent those of their affiliated organizations, or those of the publisher, the editors and the reviewers. Any product that may be evaluated in this article, or claim that may be made by its manufacturer, is not guaranteed or endorsed by the publisher.
